# Evolutionary concept analysis of health seeking behavior in nursing: a systematic review

**DOI:** 10.1186/s12913-015-1181-9

**Published:** 2015-11-27

**Authors:** Sarieh Poortaghi, Afsaneh Raiesifar, Parisa Bozorgzad, Samad E. J. Golzari, Soroor Parvizy, Forough Rafii

**Affiliations:** School of Nursing & Midwifery, Tehran University of Medical Sciences, East Nosratst, TohidSq, 1419733171 Tehran, Iran; Physical Medicine and Rehabilitation Research Center, Tabriz University of Medical Sciences, Tabriz, Iran; Students’ Research Committee, Tabriz University of Medical Sciences, Tabriz, Iran; Department of Pediatric Nursing, Nursing and Midwifery Faculty, Iran University of Medical Sciences, Tehran, Iran; Centre for educational Research in Medical Sciences (CERMS), Iran University of Medical Sciences, Tehran, Iran; Center for Nursing Care Research, School of Nursing & Midwifery, Iran University of Medical Sciences, Tehran, Iran

**Keywords:** Health seeking behavior, Concept analysis, Evolutionary method, Nursing, Systematic review

## Abstract

**Background:**

Although the research in health seeking behavior has been evolving, its concept remains ambiguous. Concept clarification, as a central basis of developing knowledge, plays an undeniable role in the formation of nursing sciences. As the initial step toward the development of theories and theoretical models, concept analysis is broadly used through which the goals can be used and tested. The aim of this study was to report an analysis of the concept of “health seeking behavior”.

**Method:**

Employing a rigorous evolutionary concept analysis approach, the concept of health seeking behavior was examined for its implications, use, and significance in the discipline of nursing between 2000 and 2012. After applying inclusion and exclusion criteria, a total of 40 articles and 3 books were selected for the final analysis.

**Results:**

The definition of attributes, antecedents, and consequences of health seeking behavior was performed through concept analysis. Core attributes (interactional, processing, intellectual, active, decision making based and measurable) were studied. The antecedents of concept were categorized as social, cultural, economic, disease pattern and issues related to health services. Health-seeking behavior resulted in health promotion and disease risk reduction. In addition, it led to predicting the future probable burden of the diseases, facilitation of the health status, early diagnosis, complete and effective treatment, and complication control.

**Conclusion:**

Health-seeking behavior, as a multi-dimensional concept, relies on time and context. An awareness of health-seeking behavior attributes antecedents and consequences results in promoting the status, importance and application of this concept in the nursing profession.

## Background

The term “Health” conveys numerous definitions which differ across cultures, different age groups, as well as people with various life experiences [1]. Every society provides a distinct definition of the terms disease and health and defines roles and activities of healthy, sick or disabled persons and adjusts the expectations and responsibilities of individuals, families and communities [2]. Furthermore, economic, social and legal variables might affect the way health or diseases are defined [3]. In general, health is the physical, mental and spiritual well-being as well as a sense of having potential energy. This general definition also implies the normal function of body tissues and organs and their adaptation with the physical and psychological environment. Thus, proper health should be well-adjusted on the basis of physical, mental and emotional capacity of individuals including their daily activities [4]. A unanimously-accepted definition of health has been provided by WHO: “health is a state of complete physical, mental and social well-being and not merely the absence of disease or infirmity” [5].

Nowadays, health and well-being increasingly occupy the front pages of newspapers and headlines among health objectives and priorities. A lot of research has shown that preventable risk behaviors such as substance abuse, unprotected sexual encounters, poor dietary and physical activity patterns, reckless driving and failure to use seatbelts significantly contribute to adolescent morbidity and mortality [6]. Many studies were conducted on these issues; however, they were not specified explicitly for the relationship between health and health-seeking behavior as well as interventions required to lessen these risks in order to achieve the goal of changing behavior. As a consequence, there is no clear definition of its concept and characteristics. [6].

Based on the reviewed literature, Health-seeking behavior is defined as an individual’s deeds to the promotion of maximum well-being, recovery and rehabilitation; this could happen with or without health concerns and within a range of potential to real health concerns [7]. There is a general consensus in both developed and developing countries that providing education and knowledge at the individual level are not sufficient per se to promote a change in behavior [8]. Understanding local perceptions of health needs, the process of health decision-making, and concerns and considerations of locals, are key components in understanding health seeking behavior in any health condition [9].

Various theories and models were developed to help understand and explain health related behavior, and suggest strategies to achieve desired behavioral change. The Health Belief Model (HBM) is one of the most widely used conceptual frameworks to explain and describe health related behaviors. Therefore, it is used as a guideline for health behavior interventions [10]. The knowledge of all of these factors is believed to be imperative to the planning process of successful interventions and the expansion of existing health services. The Theory of Planned Behavior (TPB; Ajzen 1988) and Theory of Reasoned Action (TRA; Fishbein and ajzen 1975) are considered deliberative processing models implying that people’s attitudes are formed after careful consideration of available information and the attitudes cause behavior [11]. However, there is some criticism for using these theories in action to conduct studies. Unfortunately, studies in this field often describe patterns of behavior without clarifying causes, attributes, antecedents as well as consequences for the behavior, as a result they fail to provide valuable recommendations [9, 11].

Overuse of health services in certain contexts has been highlighted in many studies; however, underuse and delay in help-seeking for serious conditions in which timely consultations could be lifesaving have also been emphasized [12]. Although numerous researches have been conducted in the field of health seeking in nursing [12–23], considering the negative effects of not seeking health behaviors such as delay in diagnosis and treatment and poor outcomes, very few efforts have been made to promote health-seeking behavior. This might be due to the lack of proper understanding of the complexity and variety of the concepts as the basic building blocks contributing immensely to the theory evolution [24]. Therefore, in-depth research is vital to visualize the real picture of the “health seeking behavior” concept.

Concept clarification, as a central basis of developing knowledge, plays an undeniable role in the formation of nursing sciences. Assessing the strengths and weaknesses of a concept and also its classification or characterization are achieved through concept clarification [24–26].

*“Concept analysis*”, as one of the main strategies in concept development, aims at understanding structure and functions of a concept, examines its basic elements and also provides the theorist, researcher or clinician with various possibilities to profoundly probe the concept. Concepts are composed of attributes; i.e., characteristics distinguishing a concept from another. Therefore, concept analysis is valuable in purifying and clarifying indefinite concepts in a theory [25].

As the initial step toward the development of theories and theoretical models, concept analysis is broadly used through which the goals can be used and tested [24–27]. The definitions of concepts are clarified through clinical studies and application of the instruments that are central to the entity of every concept [25]. Various methods have been used to analyze concepts relevant to the field of nursing science. Being affected by the context they are used in, concepts not only improve over time [24, 26] but also endure continuous dynamic changes. Hence, they redefine the context, surrogate and related terms, antecedents, attributes, examples and consequences. However, analyses simply indicate a direction toward further research yet they do not offer solid conclusions. So, Rodgers’ evolutionary approach is suitable for analyzing “health seeking behavior” concept, thanks to its dynamic context-based nature.

Knowledge generation resulting from analysis of “health-seeking behavior” would ultimately become advantageous in the establishment of health education and individualized nursing; both aiming at refining self-care abilities and health. To alter behavioral patterns and excel health practices, we need to have a thorough understanding of human behavior as policy makers in the field of health uncover the effects of human behavioral factors on the quality of health care provision. Surprisingly, not only is health-seeking behavior found rarely in commonly-used medical textbooks but also most health-seeking behavior studies do not provide any effective and practical recommendations. Consequently, a disastrous loss of medical practice and health systems development programs would be inevitable considering the fact that appropriate understanding of health seeking behavior diminishes delayed diagnoses and excels treatment compliance and health promotion strategies. Henceforth, this study is conducted with the aim to analyze the concept of “health seeking behavior” with the use of evolutionary concept analysis.

## Methods

After identifying the concept, the most important step is determining the scope and range of literature to be viewed [24]. In our study, major data bases including Pub Med, CINAHL, MEDLINE, Scopus, Springer, Ovid, Iran Medex, Magiran and SID were searched. In a preliminary search “seek health” and “health-seeking behavior” keywords were used. Later, to achieve more precise results, inclusion criteria were identified. The main criteria for inclusion in the final analysis were the literature published in English or Persian within the context of nursing and community health added as search terms from 2000 to 2012. Preliminary result of the search led to 1530 articles which considered the inclusion and exclusion criteria and duplicated items decreased to 142 cases. At this stage, articles which were not in English or Persian and were in letter, editorial or commentary format excluded from the study, as a result the number decreased to 58. Then 16 articles which did not have extra information about health seeking behavior concept or were not accessible in full text excluded from the gained mass of information.

**Fig. 1:**
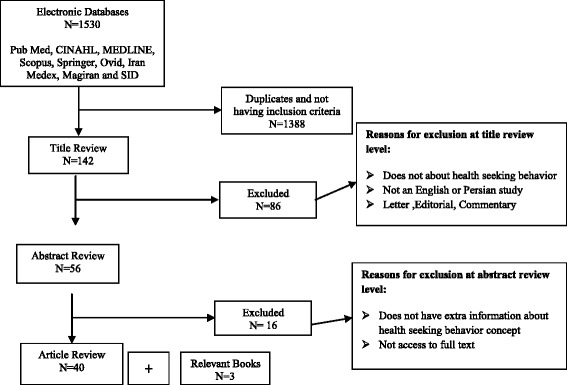
Summary of literature search and number of articles

At the final stage, 40 articles which had the term “health-seeking behavior” in their title or abstract and at the same time were available in full-text format in the field of nursing or other health sciences (including medicine and psychiatry) were selected. Meanwhile, three books were also selected because of their content coverage and their availability for authors (Fig. 1). Articles and books were reviewed vigorously by two people. Inductive content analysis of information on health-seeking behavior concept was performed by two people (first and corresponding authors) and checked by the third person (author) and themes were extracted. Information units consisted of related words, sentences, information and responses to the following questions: What are the specific attributes of health-seeking behavior? What is the definition of health-seeking behavior? Which factors are associated with health-seeking behavior? How is health-seeking behavior measured? What are the consequences or outcomes of health-seeking behavior?

**Fig. 2:**
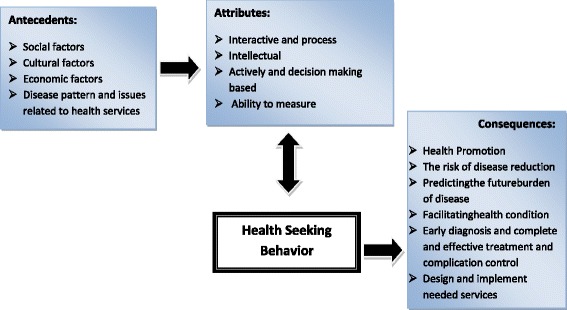
“Health-seeking behavior model”

All the textual data were inductively coded to answer the above-mentioned questions, and inter-coder agreement was assessed. A method developed by Landis and Koch [28] was used to calculate inter-coder agreement.

## Results

### Attributes

To determine the defining attributes is the primary accomplishment of the analysis that leads to the actual definition of the concept [24, 29]. Attributes of a concept include the features and permanently and clearly associated specifications that help clarification and extension of the concept depth [25]. The researchers often have to work diligently to identify the data relevant to the attributes of the concept. Actual definitions provide helpful and important data regarding the attributes. In the search for these data, researchers answer this question, “what are the characteristics of the concept?” In this study, apparent attributes of health-seeking behavior concept were interactional, processing, intellectual, active, decision-making based and measurable.

#### Interactional and processing dimension

Health seeking behavior is considered as a tool for investigating individuals’ or a population’s interaction with the health system. In other words, as health-seeking behavior is a social process, it not only involves individuals’ interactions with the social network [30] but also is a social action involving other individuals [31]. People’s interactions with the health system help to identify the factors that empower individuals to adopt ‘healthy choices’ in behaviors related to their lifestyle or medical care. Different researches have shown that health-seeking behavior is an ongoing process [31] including a logical sequence of steps beginning from the perception and evaluation of symptoms and ending with the use of different care types [30].

#### Intellectual dimension

Health-seeking behavior characterizes people’s noticeable desires for health control and their concerns about environmental impacts of the health [32]; this differs from person to person and a culture to culture [33]. Health-seeking behavior can be seen as the performance of an individual inspiring him/her to have an acceptable level of well-being; this effort is possibly due to awareness as well as to mutual cooperation of individuals and health systems. A proper understanding of human behavior is a prerequisite for behavioral and health-related changes [13].

#### Active and decision-making-based dimension

Generally, health-seeking behavior is viewed as a route through which individuals acquire information on health, illness, health promotion, and risks to health [34]. It seeks to construct healthy approaches through every day practices facilitating the feeling of ‘belonging’ via spiritual and religious practices and also the use and consumption of materials such as food and forms of treatments and therapies [35].

In other words, health-seeking behavior is the behavior of an individual with a constant health condition actively searching for ways to change his/her health habits or environment in order to move toward a higher-level wellness [32]. In addition, it is an approach through which people can monitor their body, partially distinguish symptoms and interpret them, look for medical interventions and apply other supportive resources [36, 37].

There are numerous varied definitions to health-seeking behavior as follows:

Making decisions about health choices [14], processes that affect health status [38], focus on patterns of decision-making [15], decisions and related responses [35], the expression of how people make decisions about health care and use of services [39] and an expressed or observed desire to search information for health promotion [32]. All of the above definitions imply the decision-making-based dimension of health seeking behavior concept.

#### Measurable dimension

Health-seeking behavior has been tested using quantitative (KAP studies, surveys and cross-sectional studies) as well as qualitative research methods (ethnography and Narrative studies) [13, 15, 37, 40]. This concept is mainly measured using self-reports. The health-seeking behavior questionnaire has been developed and used to study students’ and adolescents’ health status by the National Health Screening Service of Norway. This questionnaire includes three subgroups and 15 items. It is an age-sensitive scale which checks differences and comparisons within and between groups [40]. Another structured questionnaire has been used in Cambodia to compare this concept between the poor and the rich of the community, [17] it is also used in South Africa [41, 42] and Nigeria [43].

In addition, in some studies profound interviews [19, 33, 44] and focus group discussions [45, 46] or structured interviews [47] were used for data collection on health-seeking behavior.

### Health seeking behavior definitions

#### Surrogate and related terms

Identifying surrogate and related terms help to locate health-seeking behavior on nursing body of knowledge. During the analysis stages, we recognized that health seeking behavior could be interchangeable with “health-seeking decisions”, “health care-seeking behavior” or “health information seeking”.

### Antecedents

The next step in the cycle of evolutionary concept analysis is the determination of antecedents, [48] an essential section in concept analysis due to providing further elucidation about the concept of interest. Antecedents are events that have happened prior to the occurrence of the concept [26]. The antecedents of health-seeking behavior can be categorized as shown in Table 1.

**Table 1:** The antecedents of health-seeking behavior

Antecedents	Supporting references
3–1: Social factors	➢ Social networks [20, 21] and families [22] have solid roles in decision making for seeking health.
	➢ Number of other sick people and children under 5 years old in the family [22] and birth order of children [44].
	➢ Family size [14, 44]
3–2: Cultural factors	➢ Cultural beliefs about health which lead to self-care as well as using home remedies and consultation [13, 16, 19, 35, 37, 47, 51–53] as one of the barriers to health-seeking behavior
	➢ Gender inequalities exist in all communities and social classes and have negative effects on women health [13, 16, 38, 49, 54], women autonomy [17, 55] and men’s dominant role [13, 20]
	➢ Cultural preferences [15, 16]
	➢ Traditional interventions and professional attitude [37, 41, 45]
	➢ Superstition, rumors and legends [37]
	➢ Fear of stigma [45]
	➢ Previous and current perception of disease [30, 45]
	➢ Understanding the value of health [38]
	➢ Cultural taboos [51]
	➢ Negative cultural experiences such as pressure to succeed, win or physical violence [44]
	➢ Expectations of aging [12, 22, 39, 41, 56, 57]
	➢ Absence of the head of household or other key decision makers [54]
	➢ Head of household’s awareness, occupation and level of education [15, 41, 46, 49]
	➢ Ethnicity [16, 22, 56]
	➢ Marital status [22, 36]
	➢ Denial of disease, especially by women [45].
3–3: Economic Factors	➢ Family income [14, 16, 17, 20, 22, 23, 38, 41, 42, 44, 47, 51, 56]
	➢ Treatment and commute costs [13, 19, 20, 57]
	➢ Having insurance [16, 22, 51]
3–4: Disease pattern and issues related to health services	➢ Physical access to health services [12–17, 19–23, 41, 51]
	➢ Distance to health service center [14, 46, 58]
	➢ Poor performance of health services [13, 58]
	➢ Availability of drugs [13, 17, 19, 51, 58]
	➢ Can buy OTC medications without or with consulting a pharmacist [19, 47, 51] Expected quality of services [11, 15, 50]
	➢ Pluralism or existence of different health systems in a cultural setting [23, 35, 49]
	➢ Attitude toward health personnel [13, 41]
	➢ Perceived severity of illness [13, 22, 41, 42]
	➢ Knowledge and duration of illness [22]
	➢ Lack of suitable referral system [23, 32]

### Consequences

Although it is being associated with further clarifications, determining the consequences of the concept is another important step of analysis [25].

Improved income and attentiveness which arise following involvement in socioeconomic development programs definitely contribute to more rational health-seeking behaviors and consequently relative health status enhancement [49]. Burden of diseases and the elements of health-related behaviors are considered as fundamentals of health promotion. Awareness of the health behaviors prevalence seems an indispensible part for disease prevention and health promotion. Interestingly, health behaviors might be of synergistic impacts on the risk of diseases. In order to predict future disease burden in populations, evaluation of health determinants is vital [38]. Health-seeking behavior or seeking long-term and sustainable life practices and communal activeness that will facilitate a healthy state can differ from person to person and culture to culture [33]. Early diagnosis and complete treatment, better understanding of the health-seeking behavior of patients is important for effective management and control of disease [41].

Health-seeking behavior of the patients may have a direct bearing on the stage of disease at presentation, and consequently, on the overall prognosis [39].

In summary, health-seeking behavior results in health promotion, disease risk reduction, prediction of the future probable burden of the diseases, facilitation of the health status, early diagnosis, complete and effective treatment, and complication control(Fig.2).

#### Inter-coder agreement

A coding agreement analysis was conducted by two people (first and corresponding authors) The two coders identified element pairs within texts at the code and category level. An overall inter-coder agreement of 0.79 was achieved. This demonstrates “substantial agreement” as described by Landis and Koch [50].

## Discussion

The purpose of the present study was to investigate health-seeking behavior in the health science literature in order to identify its attributes, antecedents and consequences of concept. Results obtained from our study revealed that important attributes of health-seeking behavior concept are interactive and process (health-seeking behavior is a social process, it not only involves individuals’ interactions with the social network [30] but also is a social action involving other individuals), intellectual (people’s noticeable desires to health control and their concerns about environmental impacts on the health [32] which differs from person to person and culture to culture), active (actively searching for ways to change his/her health habits or environment to move toward a higher-level wellness) and decision making based (health-seeking behavior is making decisions about health choices [14] or processes that affect health status [38] as well as focusing on patterns of decision-making [15] decisions and related responses and ability to measure has been tested using quantitative (KAP studies, surveys and cross-sectional studies) as well as qualitative research methods (ethnography and Narrative studies) dimensions. Health-seeking behavior antecedents include social, cultural, and economic and disease patterns and issues related to health services. Hence, policy makers should pay great attention to provide access to health services in social, cultural and economic contexts for all society members. Consequences of such strategies would be health promotion, disease risk reduction, prediction of the diseases burden, facilitation of health support, early diagnosis, effective treatment, complications control and the last but not the least designing and implementation of the required services. The results of the concept analysis are important as they lead to integration and synthesis of the concept.

Health behavior research is pertinent to nursing practice from different aspects. Nursing consists of both interdependent and independent functions encompassing varied non-medical activities (e.g., behavior change strategies, environment manipulation and improving access to service). Nursing interventions promote the aspects diminishing poor outcomes and maximizing health. Consequently, research in the field of nursing is required in order to outline client and environmental factors that are associated with behaviors essential to the self-care and higher levels of health.

In comparison with other professionals, nurses have more frequent and longer interactions with people in most health care environments. In the meantime, it is always assumed that nurses are rational and competent with desire to take care of themselves and others. Health-seeking behavior concept analysis generates knowledge. Focusing on the improvement of self-care capabilities and health status with all its essential elements of attributes, antecedents and consequences of the concept form the bases of the strategies for developing individualized nursing interventions and health education plans. Principally, nursing is involved with retaining and improving the health of the society members through both primary and secondary health promoting interventions as well as illness management via facilitation of progress evaluation, self-monitoring and lifestyle changes.

Consequently, the health-seeking behavior concept is in relation with nursing knowledge and this concept analysis provides an initial baseline through a theoretical definition. Present analyses are limited regarding context as well as time. Therefore, continuous efforts are required to develop a conceptual framework of health-seeking behavior for current and future nursing.

Because of the complex nature of the word “concept”, it is expected to come across various definitions in different sciences’ literature, especially in nursing.

Considering the philosophical basis of evolutionary approach to analysis, the results of analysis do not provide a definitive answer to the question of what the concept is. Instead, they might provide a direction towards an additional inquiry. According to the results obtained from our study, health-seeking behavior can be explained as “a behavior through which a healthy individual intellectually makes decision about his/her health and also an endeavor to actively promote health through interaction with health system”.

## Limitation

Lack of access to all full-text articles on health-seeking behavior as well as using articles that were in English is considered the limitations of this study.

## Conclusion

Health-seeking behavior is a concept which relies on time and context. Results obtained from concept analysis define health-seeking behavior as a multi-dimensional concept.

Primary care services are the gateways to health care but health care consultation depends on individuals’ decisions to seek help. For the systems to work effectively and efficiently, it is important that such decisions are made appropriately. In addition, making appropriate decisions does require behavioral changes. Whether or not behavior is the source of poor health, malnutrition or mortality, it is now recognized as a critical part of the solution. The many cases on child mortality, maternal mortality, malnutrition, child development, and mental health have drawn attention to the behavioral solutions which require non-advanced technology and small expenses. As a consequence, being aware of health-seeking behavior attributes, antecedents and consequences will result in promoting the status, importance and application of this concept in nursing profession.
